# Attitudes of Portuguese Judges and Victim Support Professionals Toward Intimate Partner Homicide Committed by Women

**DOI:** 10.3389/fpsyg.2022.912855

**Published:** 2022-06-30

**Authors:** Mafalda Ferreira, Sofia Neves, Jorge Quintas

**Affiliations:** ^1^Faculty of Medicine, University of Porto, Porto, Portugal; ^2^Centro Interdisciplinar de Estudos de Género (CIEG - ISCSP U.Lisboa), Lisboa, Portugal; ^3^Instituto Universitário da Maia (ISMAI), Maia, Portugal; ^4^Faculty of Law, University of Porto, Porto, Portugal; ^5^Centro de Investigação Interdisciplinar da Escola de Criminologia - Crime, Justiça e Segurança, Porto, Portugal

**Keywords:** intimate partner homicide, intimate partner violence, homicidal women, attitudes, judges, victim support professionals, Portuguese

## Abstract

This investigation analyzed the attitudes of Portuguese judges and victim support professionals toward intimate partner homicide committed by women. By administering an online survey disseminated by the Portuguese Superior Council for the Magistracy and the Commission for Citizenship and Gender Equality, we found that both groups of professionals are not always aligned in their attitudes toward domestic violence and intimate partner homicide, which could jeopardize the articulation between both sectors needed for an effective preventive intervention. However, most professionals of both groups tend to disagree that women provoke their aggressors or lie about their condition as victims of domestic violence and agree that there is a need for increased security and prevention of intimate partner homicides against female and male victims when reporting domestic violence.

## Introduction

Historically, women have been the main victims of domestic violence compared to men (Moracco et al., 2010) and generally, in situations of women exerting violence on men, this happens in a position of self-defense, after long periods of abuse (Wilson and Daly, 1992; Pais, 1998; Taylor and Jasinski, 2011; Almeida, 2012; McPherson, 2019). Pais (1998, 2010) termed this type of crime “*battered homicide*,” a crime that is usually committed by women who, in the face of difficulties in abandoning an abusive relationship (e.g., economic dependence, conservative gender beliefs, lack of support from social and authorities), perceive the death of their (ex)partners as the most viable option for ending their suffering, particularly during more vulnerable emotional states, since even the end of the relationship constitutes a relevant risk factor (Campbell, 1992; Dutton and Kropp, 2000; Dobash et al., 2004). Global statistics on homicide (United Nations Office on Drugs and Crime, 2019) highlight that 82% of women compared to 18% of men are killed in the context of an (ex)intimate relationship.

Portugal still shows evidence of a patriarchal society and Judaic-Christian religious ideology after living under a dictatorship for 41 years, known as *Estado Novo* (Guimarães, 1986). These influences impact the western world at a social, cultural, and educational level that perpetuate symbolic violence against women and hegemonic masculinity (Scott, 1995; Leão et al., 2015), which also translates into the functioning of the Portuguese justice system.

The attitudes of professionals who work with victims and offenders in the context of domestic violence have proved to be essential to understand how they deal with and act toward this phenomenon. The scientific literature has demonstrated how their beliefs and gender stereotypes, the fact that it is a phenomenon that occurs mostly in the private sphere, the dynamics of the relationship between the victim and the abuser, and the difficulty of reporting the situation to the authorities influence the way these professionals perform their duties (Lila et al., 2010; Jordan et al., 2012). Even if public action against domestic violence has been reinforced by legislative changes in recent decades, namely in Portugal where the crime of domestic violence became public in 2000 and a victim status was implemented as another protection mechanism, it is crucial to improve professionals’ sensitivity when dealing with situations of victimization, namely if they are perceived as being of lower risk (e.g., psychological violence). Although the levels of violence legitimation among these professionals tend to be low (Machado et al., 2009), it is important to ensure specialized training for all professionals who work with victims of domestic violence aimed to tackle their stereotyped beliefs and foster less tolerance in the face of intimate partner violence (Lila et al., 2010; Chu and Sun, 2014). The specialization of professionals and services, as well as the universalization of the implementation of guidelines and action protocols, will also make victims feel more confident to file a complaint, promoting greater security (Felson and Pare, 2008; Sani et al., 2018).

Similar evidence was found by Coelho (2019) in a study carried out with the Guarda Nacional Republicana (GNR), a Portuguese military security force, which also demonstrated the existence of conservative beliefs by associating violence in intimate contexts with factors such as abuse of alcohol or other drugs, unemployment, poverty, and extra-marital relationships, without considering the relevance of gender issues in this analysis, even if they considered the importance and seriousness of their role and intervention in these situations. Another investigation by Casimiro (2008) pointed out that security force professionals in Portugal consider that the motivations for violence are communication and trust issues, jealousy, power, and control.

According to Mendes (2016) and Matos and Cláudio (2010), in the fields of security and justice forces, it is male professionals who most legitimize and excuse domestic violence, questioning whether there may be greater desensitization toward these cases due to their constant exposure, the high risk associated with the performance of their duties and low level of convictions, thereby reinforcing a more passive attitude toward victims (Mendes, 2016; Richards, 2020). Campos (2016) demonstrates that although there is some ambiguity in the beliefs of security forces professionals, security forces generally consider the crime of domestic violence as a serious problem.

Additionally, the literature suggests a greater need for training and awareness regarding the existence of male victims, as they recognize having difficulty in intervening with these victims and the lack of adequate services and responses in a system where their intervention is directed toward mostly female victims (Silva, 2014).

Regarding judges, little national research has been done on this topic, although some authors (Botelho and Gonçalves, 2016) state that Portuguese judges are more likely to apply more severe sanctions when victims of homicide are women, as well as when offenders use the right to remain silent. Internationally, Wingler (2013) showed that the attitudes of first-rate courts judges toward domestic violence in Missouri tended to be conservative, as they considered the husband the head of the family and were hesitant in the face of family separation, attributing the causes of the problem to social and economic problems, sometimes blaming the victims and suggesting couple’s counseling, and not believing in a positive change in the aggressor’s behavior through his arrest.

Judges also tend to assess the severity of situations of violence as more serious when there is physical violence, and less serious when it comes to psychological and emotional violence (Kafka et al., 2019). These professionals also tend to consider that men are aggressors and women are victims most commonly because the latter are more dependent, passive, and fragile, and experience symptoms of tiredness and burnout which make them more desensitized to these situations.

Faced with a situation of violence, it is also common for victims to resort to emergency medical services, which demonstrates the need to also train health professionals so that they can report, intervene, and refer them according to the victim’s needs (Stark, 2001). However, sometimes health professionals are not always aware of the occurrence of this scourge or do not feel responsible for acting in these situations, as they consider that it is not a medical problem and sometimes tend to blame victims, make moral judgments, attribute the causes of violence to intraindividual factors such as consumption of alcohol or other substances and/or the existence of psychopathology, or they feel uncomfortable asking questions of this nature because they consider it to be a private problem which can lead to double victimization and blocking an effective response to the request for help (Koss et al., 2001; Lisboa et al., 2003).

Given these results, it is crucial to train professionals from the various sectors who work with victims of domestic violence, investing in their training, demystifying their inappropriate attitudes, and creating protocols and guidelines for action (Campos, 2016) since the literature points to a lesser existence of inappropriate attitudes and a greater empathy the greater the training and education of these professionals (Matos and Cláudio, 2010; Gracia et al., 2011), which is especially important when dealing with male victims where secondary victimization and discrimination when asking for help is more evident (Hines and Douglas, 2010).

Considering the scarcity of national literature regarding the attitudes of professionals about homicide in intimate contexts perpetrated by women, we focused our literature review on studies on their attitudes toward domestic violence and on judicial decisions. In a study about the evaluation of judicial decisions in matters of intimate partner homicides conducted in Portugal (Agra et al., 2015), women were found to be the main victims of this scourge that occurs mostly in the contexts of domestic violence; however, there is a great reluctance victims’ part in reporting the crime to the authorities, associated with the fear of revictimization (Felson et al., 2005). Most of these crimes are committed in an unthinking or thoughtful manner, marked by violent emotions (Thomas et al., 2011) with the main motivations being the ending of the relationship, jealousy, and a conflict situation, mainly committed using bladed weapons and firearms (Agra et al., 2015). Regarding judicial decisions (Agra et al., 2015), in most situations, pre-trial detention was applied as a coercive measure, with most crimes being considered first-degree murders, generally because of the relationship of intimacy that existed between the perpetrator of the crime and the victim, as well as premeditation, persistence, and coldness in committing the crime, being punished with an average of 18 years and a half. Women convicted of intimate partner homicide tend to be sentenced with less severe penalties than men, with a more frequent suspended prison sentence. Similarly, Spohn (2000) found that gender is the most predictive factor in the sentence and a man is two and a half times more likely to be imprisoned than a woman.

Ventura (2018) also mentions how judicial decisions are influenced by the social stratification of the accused person’s social status, determining the weight of the punishments, and being more lenient or punitive accordingly. In her book, the author also reflects on the difficulties experienced by the victims with regard to reporting crimes, as they tend to be afraid of acts of retaliation by the aggressor or their family members and also the belief that the justice system is ineffective, coupled with the guilt and responsibility that is attributed to them by the jurisprudence (Beleza, 1990; Duarte et al., 2016) that silences them, which demonstrates that criminal reforms do not translate into a change in conservative beliefs that condition legal and social discourses, as it is possible to observe in several controversial court decisions that blame the victims and, namely for their sexuality, attenuating the abuser’s behavior. Although constitutionally the principle of equality is guaranteed, “the power of discretion attributed to the judiciary class (…) is constituted as a group of strong regulation of social and sexual roles, and with the power to classify, define, and name the experiences of the people involved (the person says that she was raped, but the court concludes that she was «coerced»; the person claims that she was a victim, but the court states that she is «offended») (…) The legal practices in the courts are heterogeneous and dependent on the personal beliefs and understandings of each magistrate.” (Ventura, 2018, p. 331).

Lastly, considering the presence of high levels of stress and burnout among professionals that have been found, specialized psychological support networks should exist for the impact of vicarious trauma (Schauben and Frazier, 1995; Burgess et al., 2017).

This investigation aims to analyze the attitudes of Portuguese judges and victim support professionals toward domestic violence and intimate partner homicide committed by women, analyzing:

1. Profile of the domestic violence victim.
2. Domestic violence victim’s protection.
3. Risk factors for the occurrence of intimate partner homicide.
4. Profile of the female intimate partner homicide offender.
5. Criminal justice system.

## Materials and Methods

An official request was sent to the Superior Council of the Magistracy and the Commission for Citizenship and Gender Equality, requesting the dissemination of an online survey about the attitudes of professionals in the area of justice as well as victim support professionals toward domestic violence and intimate partner homicide. This survey was approved by both institutions and reviewed by the Ethics Committee for Health of the University Hospital Centre of São João/Faculty of Medicine of the University of Porto and released in January 2019 and February 2020 by both entities. Data collection was completed in July 2019 and March 2020, respectively. Fulfillment of the online survey was voluntary, anonymous, and confidential, and it was not required to fill in or collect any variables that would allow the identification of participants (e.g., name, ID, email, telephone number).

The survey design was developed by the authors and qualitatively validated and filled by specialized professionals in the fields of domestic and gender violence as well as justice, through the discussion and evaluation of all items based on their professional experience and literature review, aiming to improve and clarify the various items.

The survey consists of two parts: the first comprises 12 questions about the socio-demographic characterization of the respondents, and the second part comprises 25 questions on a 5-point Likert scale (e.g., “I completely disagree; I disagree; I agree; I totally agree”), grouped into five main themes: profile of the domestic violence victim, domestic violence victim’s protection, risk factors for the occurrence of intimate partner homicide, profile of the female intimate partner homicide offender, and criminal justice system. Participants also had the possibility of not giving their opinion or not answering the question through the option “I have no opinion.” Subsequently, for the statistical and descriptive analysis of the completed surveys, we used the SPSS Statistics Software, version 26, using the Mann–Whitney test since this is a non-parametric test that allows to analyze the ordinal responses (Likert Scale) for each one of the 25 items, to assess whether there is a significant difference between the groups of professionals. A factor analysis was not performed since the items were evaluated individually and not their constructs as dimensions.

A total of 81 responses were obtained, 37 from judges and 44 from victim support professionals. Among the 37 responses of judges, 62.2% (*n* = 23) were filled out by female participants and 37.8% (*n* = 14) by male participants, with an average age of 46.4 years of age. All of them had Portuguese nationality (100%, *n* = 37), and 62.2% (*n* = 23) a married marital status.

Judges had an average of 16.4 years of service. Most respondents 43.2% (*n* = 16) were from Northern Portugal and 62.2% (*n* = 23) reported working directly with victims of domestic violence. Near half of the judges (51.4%, *n* = 19) report having specialized training about domestic violence (with an average duration of 13 h).

Most of the 44 victim support professional participants were female (90.9%, *n* = 40). They had an average age of 39.8 years of age, 95.5% (*n* = 42) had Portuguese nationality, and 34.1% (*n* = 15) presented a single marital status.

Half of the participants (*n* = 22) had a degree and 38.6% (*n* = 17) a master’s degree, 4.5% (*n* = 2) a PhD and, 6.8% (*n* = 3) completed secondary education. Most respondents (81.8%; *n* = 36) were trained in Social and Human Sciences, 9.1% (*n* = 4) in Health Sciences, 2.3% (*n* = 1) in Educational Sciences, and 60.8% (*n* = 3) in other scientific fields.

The institutions at which they work are quite varied, with an emphasis on Non-Governmental Organizations, universities, and victim support structures such as support services and shelters, exercising mainly the functions of psychologists, victim support professionals, social workers, and other coordination and management positions, with an average experience of 9.7 years.

## Results

Table 1 (see the end of the manuscript) presents a descriptive analysis by dimension related to the professional’s attitudes toward domestic violence, focusing on two dimensions: the profile of the domestic violence victim and the domestic violence victim’s protection. Regarding the profile of the domestic violence victim, we found significant differences between the responses of each of the groups (*p* < 0.001). Although most professionals from both groups tend to disagree that women provoke their aggressors or lie about their condition as victims of domestic violence, judges score higher averages, compared to victim support professionals. Additionally, most professionals from both groups, especially victim support professionals, tend to agree that women suffer more domestic violence than men and disagree with the possibility of more educated men committing less domestic violence against their (ex)partners than less-educated men. Participants from both groups tend to similarly disagree with the idea of women being as violent as men.

**Table 1:** Evaluation of attitudes of Portuguese judges and victim support professionals toward domestic violence.

	Judges (*N* = 37)	Victim support professionals (*N* = 44)	*U*	*p*
	M (SD) Md (Mean Rank)	M (SD) Md (Mean Rank)
*Profile of the domestic violence victim*				
20.Schooling negatively related to domestic violence	1.97 (0.56)	1.63 (0.62)	411.000	0.020
	2 (40.83)	2 (30.78)		
7.Women are as violent as men	2.17 (0.37)	2.12 (0.71)	592.500	0.751
	2 (36.75)	2 (35.45)		
8.Women provoke their abusers	1.87 (0.56)	1.25 (0.48)	306.000	0.000^***^
	2 (50.13)	2 (29.45)		
12.Women lie about being domestic violence victims	2.03 (0.30)	1.56 (0.68)	347.000	0.000^***^
	2 (45.48)	1 (28.90)		
16.Women suffer more domestic violence than men	3.18 (0.57)	3.61 (0.49)	456.500	0.001^***^
	3 (30.93)	4 (46.13)		
*Domestic violence victim’s protection*				
1.Women can defend themselves without recourse to weapons	2.44 (0.54)	2.56 (0.96)	582.000	0.600
	2 (34.69)	2 (37.08)		
9.Female victims of domestic violence do not ask for help because they do not want to	1.74 (0.50)	1.26 (0.44)	402.000	0.000^***^
	2 (49.51)	1 (31.35)		
14.Domestic violence must be resolved by the couple themselves	1.41 (0.55)	1.14 (0.35)	591.000	0.015^**^
	1 (45.03)	1 (35.57)		
17.Domestic violence must always be reported	3.14 (0.63)	3.56 (0.66)	535.500	0.006^**^
	3 (33.38)	4 (46.43)		
21.Coercive measures are effective in protecting victims of domestic violence	2.74 (0.44)	1.81 (0.69)	220.500	0.000^***^
	3 (51.89)	2 (27.13)		

^1^Scale from 1 (totally disagree) to 5 (totally agree), ^*^p < 0.05;

** *p* *< 0.01;*

*** *p < 0.001*.

In the dimension related to domestic violence victim’s protection, professionals from both groups, especially victim support professionals, disagree that women do not ask for help because they do not want it and that domestic violence is a problem that the couple must solve themselves. On the other hand, both groups, especially victim support professionals, mostly agree with the need to report situations of domestic violence. Also in this dimension, participants disagree that a woman can defend herself without using weapons and have concerns about the effectiveness of the coercive measures applied to domestic violence abusers when protecting their victims. This is one of the items with the greatest disparity in their responses since victim support professionals do not believe at all that the applied measures are effective in opposition to judges who demonstrate a more moderate position (even if with a tendency for disagreement).

Table 2 (see the end of the manuscript) presents a descriptive analysis by dimension related to the professional’s attitudes toward intimate partner homicide, focusing on three dimensions: risk factors for the occurrence of intimate partner homicide; profile of the female intimate partner homicide offender; and criminal justice system response The dimension related to the profile of the female intimate partner homicide offender indicated only one difference between the two groups: judges strongly disagree that reporting domestic violence to the authorities constitutes a risk factor for homicide in the context of domestic violence, compared with victim support professionals. The mean values for both groups are around the middle scale point concerning the idea that female victims of domestic violence are at greater risk of being murdered by their abusive partners compared to male victims. They also tend to not consider that domestic violence victims who have received death threats are more likely to kill their abusive partners, and strongly disagree that infidelity is a motivation to commit intimate partner homicide.

**Table 2:** Evaluation of attitudes of Portuguese judges and victim support professionals about intimate partner homicide.

	Judges (*N* = 37)	Victim Support Professionals (*N* = 44)	*U*	*p*
	M (SD) Md (Mean Rank)	M (SD) Md (Mean Rank)
*Risk factors for the occurrence of intimate partner homicide*				
13.Female victims of death threats are more likely to commit intimate partner homicide	2.33 (0.48)	2.46 (0.56)	364.000	0.318
	2 (27.67)	2 (31.60)		
15.Women at higher risk of suffering intimate partner homicide	2.92 (0.50)	3.12 (0.83)	625.000	0.137
	3 (35.86)	3 (42.62)		
19.Complaint as a risk factor for committing intimate partner homicide	2.31 (0.58)	2.67 (0.77)	491.500	0.023^*^
	2 (32.04)	3 (42.40)		
23.Infidelity as a motivation to commit intimate partner homicide	1.63 (0.49)	1.46 (0.55)	479.000	0.141
	2 (43.40)	1 (36.15)		
*Profile of the female intimate partner homicide offender*				
3.Marital homicidal women pose a danger to society	2.15 (0.65)	1.56 (0.70)	379.000	0.000^***^
	2 (47.35)	1 (30.24)		
5.Marital homicidal women tend to have a previous criminal record	1.81 (0.40)	1.65 (0.48)	483.000	0.152
	2 (37.42)	2 (32.05)		
6.Marital homicidal women tend to be repeat offenders	1.73 (0.45)	1.71 (0.51)	658.500	0.808
	2 (38.05)	2 (37.06)		
10.Victims of domestic violence with psychological problems are more likely to commit intimate partner homicide	2.48 (0.59)	2.51 (0.70)	385.500	0.761
	3 (28.76)	3 (29.99)		
11.Women tend to not premeditate intimate partner homicide	2.40 (0.50)	2.82 (0.72)	272.500	0.015^*^
	2 (23.90)	3 (33.74)		
22.Women use less physical force to kill	2.70 (0.47)	2.85 (0.56)	320.500	0.204	3 (25.93)	3 (30.29)
*Criminal justice system response*						
2.Justice system protection reduces the risk of intimate partner homicide	3.09 (0.57)	3.26 (0.85)	523.000	0.133
	3 (32.85)	3 (39.59)		
4.Victims of domestic violence who kill their abusers can be condemned for privileged homicide	2.90 (0.30)	3.00 (0.81)	403.500	0.260
	3 (28.95)	3 (32.98)		
18.Domestic violence risk assessment instruments are effective in preventing intimate partner homicide	2.42 (0.62)	2.29 (0.73)	540.500	0.519
	2 (36.56)	2 (33.72)		
24.Women should be more penalized than men for intimate partner homicide	1.36 (0.48)	1.21 (0.56)	615.500	0.063
	1 (43.40)	1 (36.15)		
25.A previous history of domestic violence should constitute a mitigating factor in cases of intimate partner homicide	3.03 (0.65)	3.55 (0.50)	396.500	0.000^***^
	3 (29.51)	4 (45.07)		

^1^Scale from 1 (totally disagree) to 5 (totally agree), ^*^p < 0.05;

** *p* *< 0.01;*

*** *p < 0.001*.

Considering the questions asked in the survey concerning the profile of the female intimate partner homicide offender, the main differences between both groups are the stronger disagreement of victim support professionals in relation to the danger these women pose to society and the lesser disagreement with the statement related to female tendencies to not premeditate the commitment of a homicide. All other items are evaluated by both groups similarly. There is a strong disagreement with the idea that these women have a previous criminal record, tend to be repeat offenders, and even if lesser accentuated, a disagreement with psychological problems as a motive to an increased probability of killing or that these women use less physical force to kill.

Regarding the justice system protection dimension, neither groups agree on the effectiveness of risk assessment instruments for preventing the homicide of domestic violence victims, but moderately agree that criminal justice protection reduces the risk of intimate partner homicide. Also, the application of effective coercive measures in order to protect the victim can be more successful the better and more effective the cooperation between both parts is since we found consistency in other responses such as the possibility that a woman can defend herself against a man’s physical aggression without resorting to weapons (e.g., firearms, bladed weapons, blunt objects) where both professional groups tend to disagree with item 2 where the influence of efficient authority protection while lowering the chances of a female victim of domestic violence killing her abusive partner is discussed.

Results show a strong opposition from both groups of participants considering the higher penalization of women compared to men in intimate partner homicides, but also a position of the two groups near the middle point of the scale concerning the possibility of the women being convicted of privileged homicide. Lastly, the possibility of a previous history of domestic violence constituting a mitigating factor in cases of intimate partner homicides is significantly more accepted by victim support professionals.

## Discussion

When women commit crimes, several factors are considered as aggravating or mitigating their sentences, namely the type of crime committed, their age, the existence of children or other dependent people, victim–offender relationship, ethnicity, education, and criminal record (Paula and Caridade, 2018). However, these variables do not have the same weight in decision-making. As suggested by Freiburger (2011) and Koons-Witt (2002), since the existence of children or other dependent people is generally a protective factor for non-recidivism, it results in a lower probability of receiving harsher sentences. Thus, the protective attitudes of judges toward those who typically play a nuclear family role, i.e., mothers, seem to favor criminal women (Daly, 1989), correlating with more lenient sentences (Freiburger, 2011). On the other hand, some authors claim that women tend to be more severely punished than men, since the system stigmatizes female offenders as double transgressors, deviating from the law and socially expected gender roles (Lombroso and Ferrero, 2013; Caridade and Nunes, 2017). Hence, keeping mothers under detention would be the best way to protect their children (Koons-Witt, 2002).

Several studies have pointed out the relevance of attitudes of professionals on female crime, aiming to understand if there is sex-based reasoning in judging female and male offenders. The application of different criteria to evaluate both seems to be influenced by stereotypes that reinforce a conservative vision of gender roles. Thus, the transgressive nature attributed to women offenders encompasses the idea of violation of expectations of traditional female roles, increasing offenders’ culpability and consequently, the severity of sentencing (Paula and Caridade, 2018).

In our study, Portuguese judges and victim support professionals reveal generally well-informed attitudes on intimate partner homicide committed by women, moving away from conservative approaches. Victim support professionals present more well-informed attitudes, especially on domestic violence and homicide dynamics. This can be explained by the mandatory specialized qualification victim support professionals must have to work with victims defined by Law number 112/2009, September 16, which regulates the juridical regimen applicable to domestic violence prevention, protection, and assistance to victims. However, the fact that Portugal has lived under a dictatorial regimen until 1974, with a very conservative culture where women owed total obedience to their husbands and were not allowed to leave the house if they were battered and the husband could even appeal to a judge that would force them to return home, still shows some influence in the current Portuguese society (Pimentel and Tamzali, 2014).

Although the Portuguese legislation considers the report of this public crime to the authorities a duty of all citizens, there is also a known increased risk this can cause for the victims (Felson et al., 2005). It is, therefore, relevant to consider the responses of both groups considering the importance of reassessment in risk assessment instruments and the need for increased security and prevention of intimate partner homicide when reporting domestic violence, namely through the implementation of more adequate and effective coercive measures to protect the victims.

Since it is stated in the literature that the death of one of the members of the (ex)couple frequently occurs as a result of the escalation of previous abuse, it is crucial to involve the justice system in the application of the coercive measures effectively necessary to protect the victim (Agra et al., 2015), as well as to invest in strategies that focus on intervening psychologically with abusers that may not be motivated for treatment, but that can have positive changes in the beliefs that trivialize and devalue intimate partner violence or that blame the victim for their aggressive behavior (Manita, 2007; Matos, 2008), and to focus on empowering victims of domestic violence by giving them the necessary tools to be able to make informed and consented decisions and choices and build their own support networks (Han, 2003), this way preventing future situations of intimate partner violence.

In situations where there is a total absence of state protection, postponing their rights to self-defense could mean a difficult or impossible defense, and this type of defense is a much more frequent context for female-perpetrated intimate partner homicide than it is for men (Felson and Messner, 1998; Corleto, 2006; Weizmann-Henelius et al., 2012). Also, according to a systematic review on female perpetrated intimate partner homicide (Mackay et al., 2018) the most common motives for committing the crime are emotional problems, retaliation, instrumental gain or control, and self-defense after being systematically victimized by intimate partner violence (Walker and Browne, 1985; Serran and Firestone, 2004; Swatt and He, 2006; Campbell et al., 2007; Caman et al., 2016). Therefore, it is important to analyze other doctrines in the literature such as the “Most Effective Defense Theory” or “Early Self-Defense” (Palma, 1990; Carvalho, 1998), as well as the possible legal framework in the situations of Privileged Homicide and Self-Defense, as both groups also seem to agree when it comes to items 4 and 24, regarding the legal framework dimension, related to the possibility of fitting cases where female domestic violence victims who killed their abusive partners into a privileged homicide legal framework, considering these circumstances as a mitigating factor. In fact, during the 1970s in the United States of America, after carrying out an investigation into cases where women, victims of domestic violence, kill their fellow aggressors and claim self-defense due to the circumstances in which the crime took place (Cutler, 1989; Easteal, 1991; Coker, 2013), the Battered Woman Syndrome (Walker, 1992) theory emerged. This syndrome was then understood as a subtype of post-traumatic stress disorder since with the constant experience of the cycle of violence, the victim can develop similar symptoms such as low self-esteem, anxiety and depression, attention, concentration and memory difficulties, conservative beliefs and submission (Walker, 1992; Manita, 2007), but this did not mean that their liability was reduced (Dutton, 1993). These psychological impacts cause the victims to lose the ability to defend themselves and escape from abusive relationships, a concept that is known as Learned Helplessness, even though they manifest the ability to cope to deal with the abuse inflicted on them (Miller and Seligman, 1975; Walker, 2009).

For both groups, homicidal women are characterized as not having a previous criminal record, no danger, mental health, and a low risk of recidivism. Moreover, both groups believe that women do not premeditate homicide and cannot defend themselves without using weapons, with victim support professionals arguing that previous history of domestic violence should constitute a mitigating factor in cases of intimate partner homicides more than judges.

As far as risk factors are concerned, both groups undervalue the relevance of previous threats and the danger associated with reporting domestic violence to police authorities. Coercive measures applied to domestic violence offenders, as well as risk assessment measures, are described as ineffective, although judges are less incisive in their valuation.

Both judges and victim support professionals assert that women should not be punished more severely than men for intimate partner homicides. The possibility of privileged homicide must be considered in cases where the homicide offenders were prior victims of domestic violence.

This research aimed to explore the social scourge of domestic violence, through a different lens to reflect on women as intimate partner homicide offenders, from the perspective of Portuguese judges and victim support professionals who work directly with this population.

We thus conclude that these different groups of professionals are not always aligned in their attitudes toward the phenomenon of domestic violence in general, and about the intimate partner homicide perpetrated by women, in particular. Thus, the cooperation between both sectors is essential for effective prevention and intervention in situations of intimate partner violence, although both groups of professionals tend to agree on important topics such as that there is an association between prior exposure to domestic violence and the perpetration of intimate partner homicide.

Most professionals of both groups tend to disagree that women provoke their aggressors or lie about their condition as victims of domestic violence, and they agree that there is a need for increased security and prevention of intimate partner homicide when reporting domestic violence since risk assessment instruments are not always effective, so there is a need to implement more adequate and effective coercion measures to protect the victims. Also, most professionals from both groups tend to agree that women are the main victims of domestic violence when compared to men and that education is a protective factor against domestic violence as well as a mitigating factor when sentencing (Paula and Caridade, 2018).

Due to the impacts caused by the violence suffered, victims should have accessible and safe ways to obtain assistance from competent authorities (Equipa de Análise Retrospetiva de Homicídio em Violência Doméstica, 2020). Due to this, and knowing that the death of one of the members of the (ex)couple tends to arise as a result of an abuse escalation, it is essential to invest in the prevention and tackling of violence in intimate contexts, namely through the specialized and active involvement of victim support networks, health systems and, in particular, justice *via* the application of the necessary coercive measures to protect the victim (e.g., removal of the aggressor), the application of risk assessment instruments (e.g., RVD-1 l), and security force procedures (Agra et al., 2015).

It is also important to tackle some conservative beliefs, a greater investment should occur in the training and awareness of these professional audiences that deal directly with these women since according to what the literature describes, the main reasons for women to kill their partners are emotional issues, revenge or self-defense after being repeatedly victimized by them (Serran and Firestone, 2004; Campbell et al., 2007; Caman et al., 2016), to raise awareness among these professionals on the need for specific training on domestic violence, and to call for greater adherence in this type of research, thus allowing the collection of data on a larger sample of participants and increasing the reliability of the findings.

One of the limitations of this study is the need to consider the social desirability that may be present in the responses of the participants when responding to the online survey, providing more politically correct answers that may not always represent their true feelings, beliefs, and attitudes toward the topic.

Given the scarcity of studies regarding female murderers, we believe this paper brings important considerations that could be complemented with future investigations about the perceptions of the justice professionals (e.g., lawyers, judges) directly involved in these types of lawsuits, as well as the society’s beliefs and media coverage regarding homicide perpetrated by men and women.

Finally, it is important to highlight the importance of gender in the analysis of crimes of homicide, namely those that occur in a context of intimacy. According to Dobash and Dobash (2017, p. 131), “Unless the murders of women are examined separately from the murders of men, that is, disaggregated by gender, little can be known about this type of murder which is otherwise lost within the larger number of male–male homicides.,” with the same holding for women and men as perpetrators.

## Data Availability Statement

The original contributions presented in the study are included in the article/supplementary material, further inquiries can be directed to the corresponding author.

## Ethics Statement

The studies involving human participants were reviewed and approved by Comissão de Ética para a Saúde do Centro Hospitalar Universitário de São João/Faculdade de Medicina da Universidade do Porto. The patients/participants provided their written informed consent to participate in this study.

## Author Contributions

MF and SN contributed to conceptualization and methodology. MF contributed to investigation and writing—original draft preparation. MF, SN, and JQ contributed to data curation. SN and JQ contributed to writing—review and editing. All authors contributed to the article and approved the submitted version.

## Conflict of Interest

The authors declare that the research was conducted in the absence of any commercial or financial relationships that could be construed as a potential conflict of interest.

## Publisher’s Note

All claims expressed in this article are solely those of the authors and do not necessarily represent those of their affiliated organizations, or those of the publisher, the editors and the reviewers. Any product that may be evaluated in this article, or claim that may be made by its manufacturer, is not guaranteed or endorsed by the publisher.
